# Ultra-Sensitive Detection of Mercury by Using Field-Effect Transistor Biosensors Based on Single-Walled Carbon Nanotubes

**DOI:** 10.3390/bios15120779

**Published:** 2025-11-26

**Authors:** Chao Lu, Qiuxiang Lv, Yuanwei Lin, Li Gao

**Affiliations:** 1 Department of Medical Imaging, Jiangsu University Affiliated People’s Hospital (Zhenjiang First People’s Hospital), Zhenjiang 212002, China; 2 School of Life Sciences, Jiangsu University, Zhenjiang 212013, China; 3 School of Life Sciences, Qinghai Normal University, Xining 810000, China

**Keywords:** Hg^2+^ detection, field-effect transistor biosensors, high sensitivity, single-walled carbon nanotubes, DNA sequence

## Abstract

In recent years, the amount of mercury discharged by human activities has continued to increase. Most of the mercury in surface water settles into the sediment, where it can be directly or indirectly transformed into mercury ion (Hg^2+^) compounds (such as dimethylmercury) under the action of microorganisms. Hg^2+^ display high toxicity and bioaccumulation in food, such as fish and rice, and thus the contamination of mercury ion is a serious concern for human health. Practical Hg^2+^ detection methods are usually limited by the sensitivity and selectivity of the used methods, such as colorimetric determination and fluorescence biosensor based on the solution phase. Therefore, it is urgent to develop Hg^2+^ detection methods in the practical environment with high sensitivity and selectivity. DNA is low-cost, relatively stable, and has been used for different fields. In this study, DNA for Hg^2+^detection was absorbed on the surface of single-walled carbon nanotubes (SWNTs) by using 1,5-diaminonaphthalene (DAN) based on field-effect transistor (FET) biosensors. The interaction between DNA and Hg^2+^ can be directly converted into electrical signals based on the SWNTs biosensors. The experimental results showed that the limit of detection (LOD) of Hg^2+^ without the phase-locked amplifier was about 42.6 pM. The function of the phase-locked amplifier is to achieve fast detection of the biosensor with strong anti-noise ability. Intriguingly, the sensitivity of the biosensor combined with a phase-locked amplifier to detect Hg^2+^ was further improved to be 5.14 pM compared with some current methods of biosensors. Furthermore, this biosensor has an excellent selectivity and practical detection in tap water, which demonstrates its high performance and low cost in practical application in Hg^2+^ detection. These results show this method for Hg^2+^ detection using SWNTs biosensors with a phase-locked amplifier is promising.

## 1. Introduction

In recent years, the amount of mercury discharged by human activities has continued to increase, causing mercury pollution in water bodies, mainly from wastewater emissions from industries such as chlor-alkali, plastics, and electronics. Most of the mercury in surface water settles into the sediment, where it can be directly or indirectly transformed into mercury ion (Hg^2+^) compounds (such as dimethylmercury) under the action of microorganisms. Mercury displays high toxicity and long-lasting bioaccumulation in ecosystems. Excessive mercury in the human body can cause lung injury, vomiting, diarrhea, nausea, movement disorders, as well as language and hearing impairment. Moreover, mercury damages nerves and other organs and causes severe dysfunctions such as kidney and muscle issues, deformed limbs, paralysis, degeneration and necrosis of brain cells, difficulty in swallowing, and death [1,2,3,4]. This has raised public awareness of the dangers posed by Hg^2+^. The limits in Hg^2+^ content for different fields were set. The highest permitted level of mercury in industrial pollutants, according to the regulations of the European Parliament and Council of the European Union, is 0.03 mg⋅mL^−1^ (150 nM) [5]. Therefore, developing advanced methods for Hg^2+^ detectionin the practical environment with high sensitivity and selectivity were further required [6].

At present, some techniques have been established to detect Hg^2+^ in the literature, including but not limited to atomic absorption spectrometry (AAS) [7], mass spectrometry (MS) [8], and atomic emission spectrometry (AES) [9]. However, some methods either have poor detection sensitivity or require expensive equipment, complicated processes, and lengthy pre-treatment. DNA is low-cost, relatively stable, and has been used in different fields. And DNA sequence with ‘T’ bases can specifically bind with Hg^2+^. To address these issues for Hg^2+^ detection, DNA sequence was used to build biosensors for Hg^2+^ detection with convenience offabrication and measurement [10]. However, the actual sensitivity of the detection based on biosensors based on the DNA sequence also needs to be improved. A field effect transistor (FET) biosensor is mainly made of nano-materials or organic semiconductors (OSC) as channel sensing film, such as carbon nanotubes, graphene, and pentacene [11,12,13]. A metal oxide semiconductor (MOS) FET device usually consists of the source, drain, and gate terminals, where the current in the semiconductor channel between the source and drain (*I_D_*) terminals is regulated by the electronic field generated by the voltage applied between the gate terminals (V_G_) and the source drain (V_SD_) terminals. Gate voltages can be conducted through either the bottom gate, named ‘back gate FET’, or the top gate, named ‘top gate FET’ [14]. FET biosensors have been widely used in the biological field due to their characteristics of high selectivity; high sensitivity; real-time response; and unlabeled detection in the detection of proteins, nucleic acids, and viruses [15,16,17,18]. Single-walled carbon nanotube (SWNT) is a typical carbon nanomaterial with high quality and preparation convenience compared with some other carbon nanomaterials like fullerene and graphene. Nonetheless, SWNT-based FET biosensors are limited by the absence of functional groups on the surface of the SWNTs and the poor electrical connectivity between the molecular detector and SWNTs. These issues can be further solved by using a connecting compound and a lock-in amplifier.

A lock-in amplifier is an electronic device that can measure dynamic signals in real time with strong anti-noise ability; it is mainly composed of oscillators, mixers, low-pass filters, and other parts. For weak signals buried in noise, the principle of orthogonality is used to retain the signal of selected frequency, weaken the impact of noise, and accurately extract the signal through frequency conversion [19,20]. Moreover, the other function of the phase-locked amplifier is to achieve fast detection of the biosensor because the phase-locked amplifier can provide a fast response to the signals. In this study, 1,5-diaminonaphthalene (DAN) as a connecting channel between the surface of the SWNTs and DNA sequence and a lock-in amplifier as the measurement instrument were used to improve the sensitivity of Hg^2+^ detection. Then, SWNT biosensors were further used for detection in real samples.

## 2. Materials and Methods

### 2.1. Chemical Reagents and Experimental Materials

DNA, 5′-NH_2_-TTT TTT GGG TGG GTG GGT GGG TTT TTT-3′, was from Shanghai Sangon Co., Ltd. (Shanghai, China) and was verified for correctness via high performance liquid chromatography (HPLC) purification and mass spectrometry analysis. The ultrapure water (UP) used for HPLC was prepared using a laboratory ultrapure water system (UPF-10 L). Other reagents such as ferric chloride (FeCl_3_), sodium chloride (NaCl), calcium chloride (CaCl_2_), potassium chloride (KCl), zinc chloride (ZnCl_2_), magnesium chloride (MgCl_2_), concentrated sulfuric acid (H_2_SO_4_), hydrogen peroxide (H_2_O_2_), and ethanol (CH_3_CH_2_OH) were purchased from Sinopharm Chemical Reagent Co., Ltd. (Shanghai, China). Copper mesh AG200F4 was purchased from Medium Lens Instrument Co., Ltd. Dithiothreitol (DTT) was purchased from Sigma-Aldrich (Shanghai, China). Silicon wafer withthermal oxide layer on itand gold thread were purchased from Hefei Kejing Co., Ltd. (Hefei, China).

### 2.2. Instruments

The tubular high temperature vacuum muffle furnace (OTF-1200X) equipped with three mass flow controllers (SSL-3Z-LCD) was purchased from Hefei Kejing Technology Co., Ltd. (Hefei, China). The high vacuum resistance thermal evaporator (ZHD300) was made by Beijing Taikenuo Technology Co., Ltd. (Beijing, China). Vacuum dryer (IPC250) was made by Baoding Fage Instrument Manufacturing Co., Ltd. (Baoding, China). The vacuum systems mentioned above were maintained by a two-stage vacuum pump (VRD-8/220V/50/60Hz). The room temperature magnetic stirrer (B11-3) was purchased from Shanghai Sile Instrument Co., Ltd. (Shanghai, China). Fourier transform infrared spectroscopy (FT-IR) was conducted by a Nicolet iN10 (Shanghai, China). The field emission scanning electron microscope (FE-SEM) withultra-high resolution was made by Hitachi (SU8030) (Beijing, China). The semiconductor parameter meter (B1500A) was purchased from Keysight Technologies (Beijing, China), and the lock-in amplifier (MF-DEV5811) was purchased from Beijing Yanjing Electronics Co., Ltd. (Beijing, China). The BioTek Synergy H_4_ multipurpose microplate reader was used to measure the fluorescence intensity. Origin 8.0 was used to process the data.

### 2.3. Principle of the Biosensor

FET based on SWNT was used as an ultra-sensitive biosensor for Hg^2+^ detection, and multi-T DNA sequences were functionally attached to the surface of the SWNTs to selectively determine Hg^2+^. A thermal layer of SiO_2_ on the wafer surface allows for thesubstrate insulation and can be used as the dielectric layer in the FET device. Therefore, the SWNT connected to the substrate is the unique conductive channel between the source and the drain, which constitutes the SWNT FET with the back gate type and low leakage current. Because charged biomolecules are adsorbed to SWNTs, the band structure of SWNT FET can be modified by the biomarkers, resulting in changes in source-drain current (*I*_D_). Therefore, such changes in electrical properties of the devices can be used to detect biomolecules.

**Figure 1:**
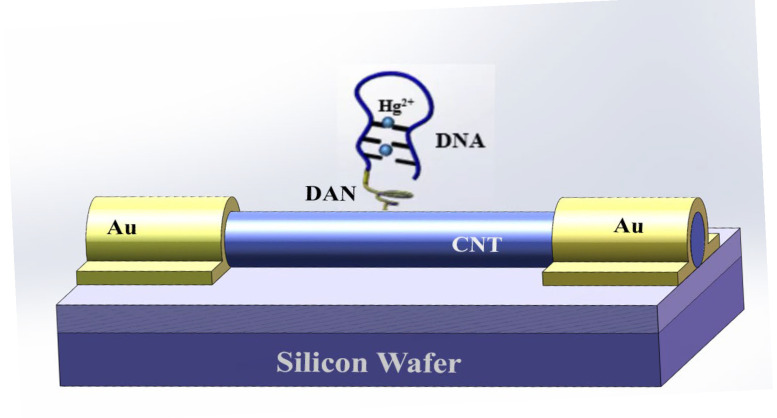
Schematic diagram of field-effect transistor biosensors using single-walled carbon nanotubes. DNA sequences with ‘T’ bases were functionally attached to the surface of single-walled carbon nanotube to selectively determine Hg^2+^. The conformation of DNA sequence change and the corresponding current change were induced. Au was gold electrode; DAN was 1,5-diaminonaphthalene; CNT was carbon nanotube in this figure.

Due to the π-π stacking interaction between the coagulant DAN and the SWNTs sp^2^ plane, DAN can be connected to the SWNTs. Thus, the SWNT FET biosensor was treated with 10 μM DAN in methanol (40 μL) at room temperature for 3 h and then washed with 0.01 M phosphate buffer solution (PBS, pH 7.4). After that, 40 μL 2% (V/V) glutaraldehyde (GA) was added for 3 h to bind DAN by Schiff reaction. Then, 40 μL ssDNA (20 μM) was fixed to GA on the surface of the SWNTs at room temperature for 6 h after the biosensor was rinsed with PBS buffer to remove excess GA. Its electrical characteristics were measured by the semiconductor parameter meter to screen qualified devices. DNA is conductive. As shown in Figure 1, the DNA sequence was connected to the surface of the SWNTs. Hg^2+^ can react with the DNA sequence with the stable structure of “T-Hg-T”. Therefore, the conformation of DNA sequence change and the corresponding current change were induced. Finally, different concentrations of Hg^2+^ onto the surface of the SWNTs were added and reacted with the DNA sequence at room temperature for 30 min. The specific detection of Hg^2+^ using the SWNT FET biosensor was measured. The combination of Hg^2+^ and DNA sequence resulted in DNA conformation change, which can further lead to the static change at the interface and electrical signal change.

### 2.4. Single-Walled Carbon Nanotube Preparation

**Figure 2:**
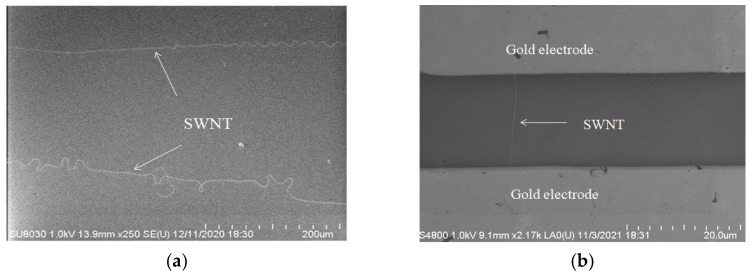
(**a**) Scanning electron microscope image of single-walled carbon nanotubes. (**b**) Scanning electron microscope of single-walled carbon nanotubes between gold electrodes. SWNT was single-walled carbon nanotube in this figure.

SWNTs were prepared by chemical vapor deposition (CVD). In this method, SWNTs were grown by the chemical reaction of two or more precursors through a template attached with catalyst particles at 800~1200 °C. First, concentrated H_2_SO_4_ and H_2_O_2_ liquid mixture with a volume ratio of 7:3 was added to the petri dish. Then, a sliced silicon wafer of about 1 cm^2^ was added, and the above system was heated to 110 °C for 2 h. After that, the H_2_SO_4_ and H_2_O_2_liquid mixture in the dish was removed, and the waferwas soaked by ultra-pure water with the help of ultrasonic cleaning. After 10 min, the silicon wafer was taken out and cleaned by ultra-pure water again. Finally, it was blow-dried with nitrogen and placed under vacuum for later use. The silicon wafer spin-coated with 0.05 mmol/L Fe(OH)_3_ was put into the muffle furnace on the quartz boat and placed in the middle of the muffle furnace chamber. Second, the vacuum was kept and maintained in the furnace, and argon gas (Ar, 212 sccm) was injected under the monitoring of mass flow controller. Third, hydrogen (H_2_, 297 sccm) was then introduced. Ten minutes later, the carbon source—ethanol (argon as carrier gas)—was introduced. After 20 min, ethanol, hydrogen, and argon were gradually shut down, and the muffle furnace began to cool down. Figure 2a shows the scanning electron microscope (SEM, Hitachi SU8030, Beijing, China) image of the SWNTs.

### 2.5. Thermal Evaporation of Metal Electrode

Then 80 nm Cr and 200 nm Au were thermal evaporated on the silicon wafers. The purpose of Cr evaporation was used to make the silicon wafer have better contact with the gold electrode. As shown in Figure 2b, the SEM image of the SWNT after gold electrode evaporation demonstrated that SWNT spanned the gold electrodes, showing that the SWNT FET sensors had been made.

### 2.6. 1,5-Diaminonaphthalene Connection

After the SWNT FET biosensor was successfully prepared, it was treated with DAN dissolved in methanol (10 mM, 40 μL) at room temperature for 3 h and then washed with 10 mM PBS (pH 7.4) to remove the excess DAN. Then, 40 μL 2% (V/V) glutaraldehyde (GA) was added and stayed for 3 h. After combination with DAN via the Schiff reaction, the excess GA was washed by PBS buffer. Then the DNA sequence was connected on the surface of SWNT via DAN.

### 2.7. DTT Reaction with Hg^2+^

DTT is a sulfhydryl compounded with sulfhydryl groups at both ends, which is often used as a small molecule reducing agent [21]. Sulfur has low electronegativity and high polarizability, while Hg^2+^ has a large volume and low positive charge. Sulfhydryl has strong adsorption for Hg^2+^. After adding 100 μM Hg^2+^on the surface of the SWNT FET electrode and then immersing it in 2.5 mM DTT/ethanol solution, the SWNT biosensors were kept for 24 h at 4 °C. After cleaning with ultra-pure water and drying with nitrogen, the current of the device was measured. Then, 100 µM Hg^2+^ was added again and the reaction time was for 30 min at room temperature. After adding Hg^2+^, the current of SWNT FET decreased due to the combination of “T-Hg^2+^-T” with DNA. After adding DTT, the sulfhydryl group absorbed Hg^2+^ with positive charges and enhanced the electronic conductivity of SWNT FET on its surface, which restored the current.

## 3. Results and Discussion

### 3.1. Current Changes Before and After Connection of DNA

**Figure 3:**
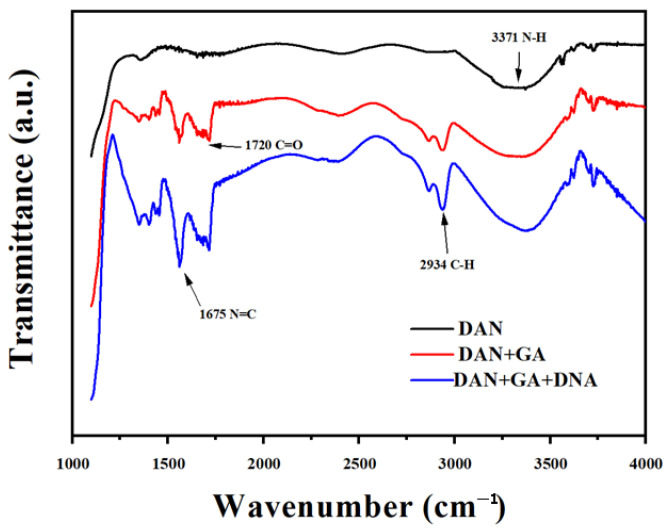
FT-IR spectrums of DAN, DAN+GA, and DAN+GA+DNA. NH_2_ and –C=O are the characteristic functional groups of DAN and GA+DAN, respectively. C=N is the characteristic functional group of DAN+GA+DNA. Thus, it is shown that DNA was connected to DAN successfully.

**Figure 4:**
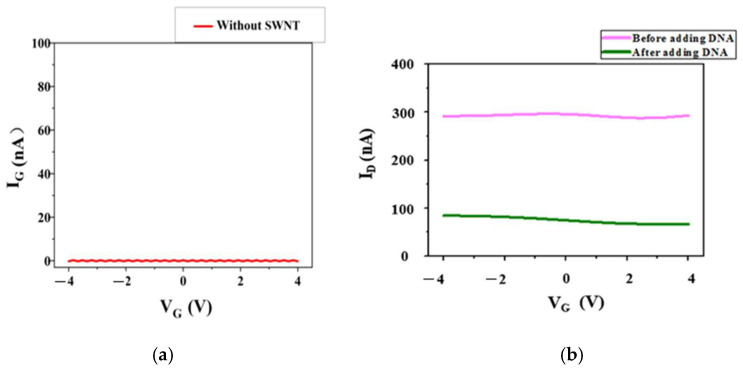
(**a**) The control device without the single-walled carbon nanotubes showed no current value because the single-walled carbon nanotubes in the sensors were the same as the conductive material. (**b**) The currents for the biosensor before and after connecting DNA on the surface of single-walled carbon nanotubes. After the addition of DNA, *I*_D_ was significantly decreased. This showed that the DNA sequence was connected on the surface of single-walled carbon nanotubes and also decreased the current value of biosensor.

Usually, the surface of SWNT has no functional groups. The Schiff-base reaction proceeded through chemical attachment between the aldehyde group of GA and the amine group of the DAN connected at the SWNT. DNA was immobilized to the GA on the SWNT surface, which was also based on the Schiff-base reaction [22]. As shown in Figure 3, it is known that -NH_2_ and –C=O are the characteristic functional groups of DAN and GA+DAN, respectively. C=N is the characteristic functional group of DAN+GA+DNA. Figure 3 shows the FT-IR spectrums of DAN, DAN+GA, and DAN+GA+DNA, which demonstrate that DNA was connected to DAN successfully. Then, 40 μL ssDNA (20 μM) was fixed on GA on the surface of the SWNTs at room temperature for 6 h. After that, the excess DNA was rinsed with PBS buffer. As shown in Figure 4a, the control device without the single-walled carbon nanotubes showed no current value because SWNTs in the sensors were the same as the conductive material. As shown in Figure 4b, after the addition of DNA on the surface of the SWNTs, *I*_D_ was significantly reduced for about 200 nA. This reveals the successful conjugation of DNA on the surface of the SWNTs.

### 3.2. Hg^2+^ Sensitivity Detection

**Figure 5:**
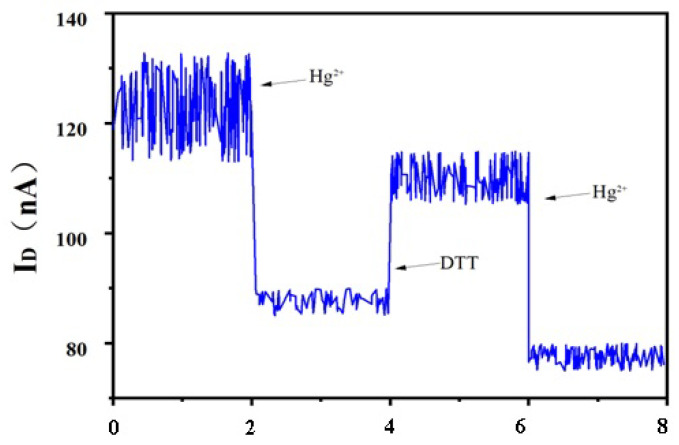
Response of SWNT FET biosensor after adding DTT and 100 µM Hg^2+^. When DTT was added to the system, the thiol (-SH) reactive groups present in the DTT molecular structure played a key role. These SH groups can quickly and efficiently adsorb Hg^2+^ bound to the DNAdue to their strong coordination ability. After Hg^2+^ was largely removed, the electron conduction pathways that were previously blocked by Hg^2+^ binding to DNA wererestored.

After the successful fabrication of the biosensor, 100 µM Hg^2+^ was added to the surface of the electrode modified with DTT, and then the current change of the biosensor was measured and calculated. The reaction between Hg^2+^ and DNA forming “T-Hg^2+^-T” led to the decrease of the measured current (Figure 5). When Hg^2+^ was added to the system, it specifically interacted with DNA sequences rich in multiple ‘T’ bases due to its unique chemical properties. This can form a structurally stable and orderly ‘T-Hg^2+^-T’ hairpin complex. The special structure interfered with the electron transport in the relevant pathway, leading to a significant decrease in the measured current intensity. When DTT was added to the system, the thiol (-SH) reactive groups present in the DTT molecular structure played a key role. These SH groupscan quickly and efficiently adsorb Hg^2+^ bound to the DNA due to their strong coordination ability. As Hg^2+^ was largely removed, the electron conduction pathways that were previously blocked by Hg^2+^ binding to DNA responded. This significantly enhanced the electron transport performance of the SWNT biosensors and resulted in a noticeable recovery of the current intensity. After adding Hg^2+^ and DTT, the change in current can repeatedly appear. However, the conductivity of the SWNT biosensor was effectively reduced due to multiple repetitions. This can decrease the current value of the SWNT biosensor.

**Figure 6:**
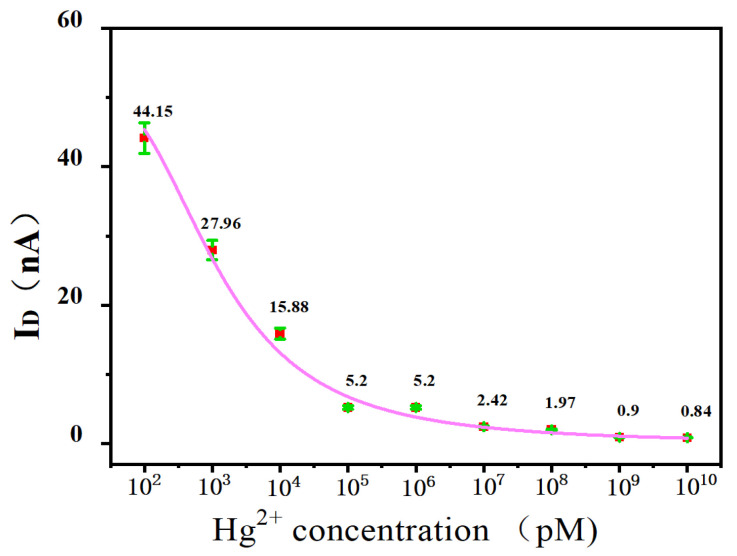
Sensitivity of field-effect transistor biosensor based on single-walled carbon nanotube to different concentrations (100 pM, 1 nM, 10 nM, 100 nM, 1 μM, 10 μM, 100 μM, 1 mM, and 10 mM) of Hg^2+^. As the binding between T-Hg^2+^-T was tight with the increasing of Hg^2+^ concentration, more Hg^2+^ bound to the device. Therefore, the current (the values were also showed in the figure) response continued to decrease (i.e., the measured *I*_D_ will continue to decrease).

Then, different concentrations of Hg^2+^ (100 pM, 1 nM, 10 nM, 100 nM, 1 μM, 10 μM, 100 μM, 1 mM, and 10 mM) were added to the SWNT FET biosensor and reacted at room temperature for 30 min. The electrical measurement results were shown in Figure 6. As the binding between T-Hg^2+^-T was very tight, with the increase of Hg^2+^ concentration, more Hg^2+^ bound to the device. Thus, the current response will continue to decrease (i.e., the measured *I*_D_ will continue to decrease). When the concentration of Hg^2+^ was in the range of 0.1~100 nM, it showed an obvious linear relationship, and its linear regression equation can be given as follows: *y* = −11.77 lg (*x*) + 52.19 with the square of thelinear correlation coefficient *R*^2^=0.99. Based on 3S/*N*, the limit of detection (LOD) was calculated to be 42.6 pM.

### 3.3. Hg^2+^ Sensitivity Detection with Phase-Locked Amplifier

**Figure 7:**
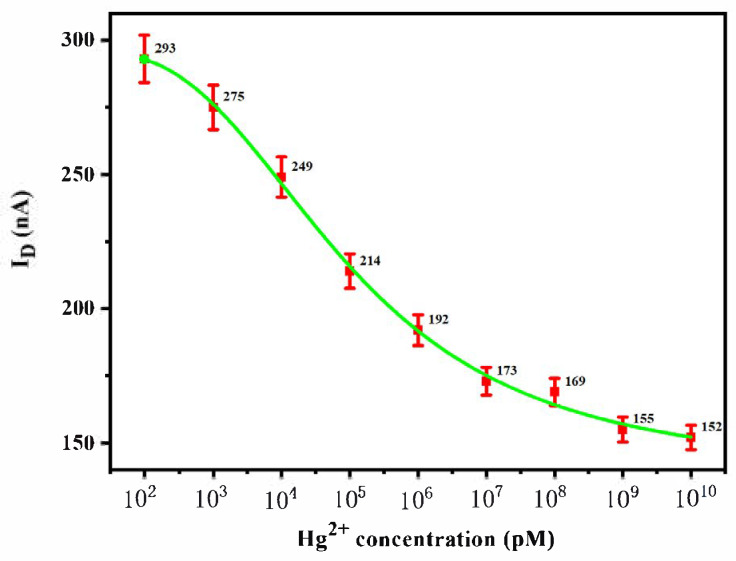
Sensitivity for different concentration of Hg^2+^ (100 pM~0 mM) based on SWNT FET biosensor by phase-locked amplifier. When the concentration of Hg^2+^ (cHg^2+^) was in the range of 0.1~1 μM, *I*_D_ (the values were also showed in the figure) had a significant linear relationship with cHg^2+^. The LOD was calculated to be 5.14 pM.

The sensitivity of the SWNT FET biosensor was further analyzed using the phase-locked amplifier to optimize the LOD. After adding different concentrations of Hg^2+^ (100 pM~0 mM) to the SWNT FET biosensor and reaction at room temperature for 30 min, its current change was detected by the phase-locked amplifier. As shown in Figure 7, when the concentration of Hg^2+^ (cHg^2+^) was in the range of 0.1~1 μM, *I*_D_ had a significant linear relationship with cHg^2+^. The linear regression equation was as follows: *y* = −25.69 lg (cHg^2+^) + 322.89, with *R*^2^ = 0.99. Based on 3*S*/*N*, the LOD was calculated to be 5.14 pM.

To compare the results of this work and the other reported literature, the linear range and the LOD of this work and some other studies are shown in Table 1. It can be clearly concluded that the LOD of this work has an obvious advantage compared with that of other works.

### 3.4. Selectivity Analysis

**Figure 8:**
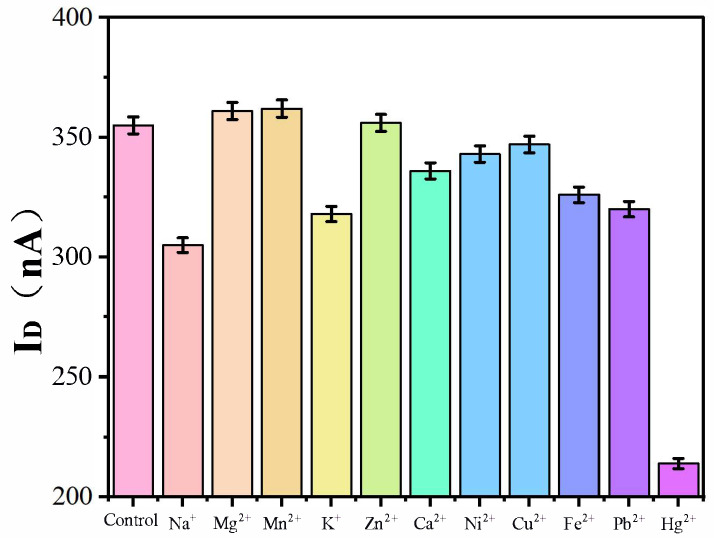
Selectivity for various metal ions (Na^+^, Mg^2+^, Mn^2+^, K^+^, Zn^2+^, Ca^2+^, Ni^2+^, Cu^2+^, Fe^2+^, Pb^2+^, and Hg^2+^) using the field-effect transistor biosensor based on single-walled carbon nanotube. *I*_D_ measured by adding Hg^2+^ was significantly lower than that of other ions. This showed that the field-effect transistor biosensor based on single-walled carbon nanotubehad good selectivity to Hg^2+^.

Selectivity is the result of strong binding by species of interest and less binding by species of noninterest, which is an important index to evaluate the specific performance of the SWNT FET sensor in detecting Hg^2+^. To assess the specificity of the biosensor for Hg^2+^ detection, Na^+^, Mg^2+^, Mn^2+^, K^+^, Zn^2+^, Ca^2+^, Ni^2+^, Cu^2+^, Fe^2+^, Pb^2+^, and Hg^2+^ were added separately at the same concentration of 100 μM to the biosensor for evaluating the selectivity of the device. As shown in Figure 8, the *I*_D_ measured by adding Hg^2+^ was significantly lower than that of other ions and control. Other ions had less change for *I*_D_. This showed that SWNT FET biosensor had good selectivity to Hg^2+^.

### 3.5. Detection in Real Samples

In order to evaluate the sensor’s stability and practical applicability and determine the practical application of the Hg^2+^ biosensor, three samples of tap water containing 100 nM, 1 nM, and 10 nM of Hg^2+^ (adding different qualities of Hg^2+^ solution into tap water) were analyzed using the SWNT FET biosensor system by the standard sampling method, and the results were shown in Table 2 below. The reproducibility of all samples was 95.98~102.05%, and the relative standard deviation (RSD) was 3.08~5.06%, which showed that the biosensor had good stability and fulfilled the requirements of practical application.

## 4. Conclusions

A SWNT FET-based biosensor was developed with single-stranded DNA for Hg^2+^ detection. The biosensor can directly convert the interaction between the DNA sequence and Hg^2+^ into electrical signals, which can maintain high sensitivity and selectivity. When the concentration of Hg^2+^ was within the range of 0.1~100 nM, an obvious linear relationship between the concentration of Hg^2+^ and the electrical signal was observed. The LOD was 42.6 pM. The environmental noise generated by its non-specific binding was reduced in order to achieve efficient signal transmission using the characteristics of lock-in amplifier. The sensitivity of the biosensor combined with a lock-in amplifier to detect Hg^2+^ was therefore further improved. The device showed a detection limit as low as 5.14 pM with excellent selectivity at the same time. DTT has strong adsorption with Hg^2+^, which can significantly enhance the electron transport performance of the SWNT biosensors and result in a noticeable recovery of the current intensity. In addition, the SWNT FET biosensor with a lock-in amplifier can be expected to be used for the detection of other biomolecules due to its simple manufacturing process and excellent sensing performance, which has great application prospects. These results show that this method for Hg^2+^ detection using SWNTs biosensors with a phase-locked amplifier is promising.

## Figures and Tables

**Table 1:** A comparison of our method with other methods for Hg^2+^ detection.

Method	Linear Range	DD OD	Reference
Imprinting sensor	0.01~100,000 nM	0.006 nM	[23]
Fluorescence sensor using metal–organic framework probe	10–60 nM	1.62 nM	[24]
Fluorescence sensor based on graphene oxide sensor	2~ 20 μM	40 nM	[25]
Electrochemiluminescence sensor	0.02 μM~0.1μM	2.52 nM	[26]
Fluorescent sensor using gold nanoparticles	1 uM~1nM	4.71 nM	[27]
Electrochemical impedance spectroscopy aptasensor	100–900 nM	5.59 nM	[28]
SWNT FET sensor	0.1~100 nM	5.14pM	This study

**Table 2:** Detection results of Hg^2+^ biosensor in tap water.

Samples	Added (nM)	Obtained (nM)	Recovery (%)	RSD (%)
1	0.1	0.102	102.05	4.22
2	1	0.98	97.81	3.08
3	10	9.60	95.98	5.06

## Data Availability

The data supporting this study’s findings are available from the corresponding author upon reasonable request.
